# Nutritional Strategies in the Rehabilitation of Musculoskeletal Injuries in Athletes: A Systematic Integrative Review

**DOI:** 10.3390/nu15040819

**Published:** 2023-02-05

**Authors:** John E. Giraldo-Vallejo, Miguel Á. Cardona-Guzmán, Ericka J. Rodríguez-Alcivar, Jana Kočí, Jorge L. Petro, Richard B. Kreider, Roberto Cannataro, Diego A. Bonilla

**Affiliations:** 1 Grupo de Investigación NUTRAL, Facultad de Ciencias de Nutrición y Alimentos, Universidad CES, Medellín 050021, Colombia; 2 Research Division, Dynamical Business & Science Society—DBSS International SAS, Bogotá 110311, Colombia; 3 Department of Education, Faculty of Education, Charles University, 11636 Prague, Czech Republic; 4 Research Group in Physical Activity, Sports and Health Sciences (GICAFS), Universidad de Córdoba, Montería 230002, Colombia; 5 Exercise & Sport Nutrition Laboratory, Human Clinical Research Facility, Texas A&M University, College Station, TX 77843, USA; 6 Galascreen Laboratories, Department of Pharmacy, Health, and Nutritional Sciences, University of Calabria, 87036 Rende, Italy; 7 Sport Genomics Research Group, Department of Genetics, Physical Anthropology and Animal Physiology, Faculty of Science and Technology, University of the Basque Country (UPV/EHU), 48940 Leioa, Spain

**Keywords:** sports injury, musculoskeletal pain, nutrients, dietary supplements, sports nutrition, sports nutritional physiological phenomena, athletic injuries

## Abstract

It is estimated that three to five million sports injuries occur worldwide each year. The highest incidence is reported during competition periods with mainly affectation of the musculoskeletal tissue. For appropriate nutritional management and correct use of nutritional supplements, it is important to individualize based on clinical effects and know the adaptive response during the rehabilitation phase after a sports injury in athletes. Therefore, the aim of this PRISMA in Exercise, Rehabilitation, Sport Medicine and Sports Science PERSiST-based systematic integrative review was to perform an update on nutritional strategies during the rehabilitation phase of musculoskeletal injuries in elite athletes. After searching the following databases: PubMed/Medline, Scopus, PEDro, and Google Scholar, a total of 18 studies met the inclusion criteria (Price Index: 66.6%). The risk of bias assessment for randomized controlled trials was performed using the RoB 2.0 tool while review articles were evaluated using the AMSTAR 2.0 items. Based on the main findings of the selected studies, nutritional strategies that benefit the rehabilitation process in injured athletes include balanced energy intake, and a high-protein and carbohydrate-rich diet. Supportive supervision should be provided to avoid low energy availability. The potential of supplementation with collagen, creatine monohydrate, omega-3 (fish oils), and vitamin D requires further research although the effects are quite promising. It is worth noting the lack of clinical research in injured athletes and the higher number of reviews in the last 10 years. After analyzing the current quantitative and non-quantitative evidence, we encourage researchers to conduct further clinical research studies evaluating doses of the discussed nutrients during the rehabilitation process to confirm findings, but also follow international guidelines at the time to review scientific literature.

## 1. Introduction

Currently, elite athletes are subjected to a grueling competitive calendar [1] which is generally associated with a higher training volume and competition load [2]. The consequence of this competitive model is not only reduced performance, as has been reported in several sports [3,4,5,6], but also an increase in the occurrence of lesions. In recent years, studies on injury prevention [7] along with the technologies and strategies to prevent them have increased exponentially; however, the incidence of sport-related injuries has remained constant [8]. An injury episode can be expressed as the number of injuries that the athlete may suffer per 1000 h of exposure to the risk of injury, both in training and in competition. It is estimated that an average of 3 to 5 million sports injuries occur in a year [9], with the prevalence being higher during competitions (72.2%) than during training (21.8%) [10]. For instance, Dupont et al. [11] reported a 6.2 times higher injury rate in soccer players who played two games a week compared to those who played only one, with the majority of injuries (76%) caused by overuse. In general, the injury rate in soccer is mostly significant during games/matches (9.5 to 48.7 injuries/1000 h in competitive male youth players, 2.5 to 8.7 injuries/1000 h in male professional players, and 12.5 to 30.3 injuries/1000 h in female players) [12]. Importantly, it has been reported that approximately 81 per 1000 elite athletes suffered an injury during competition at World Championships with a 40.9% prevalence of musculoskeletal injuries [13]. Similarly, a college basketball player has a rate of 9.9 injuries per 1000 h of competitive games, while only 4.3 injuries are sustained per 1000 h of training [14]. In the National Basketball Association professional league, the exposure rate per player is 3.26 injuries per 1000 h of competitive play, with the prevalence being higher in the first month of the league [15].

The number of musculoskeletal injuries and illnesses suffered by athletes during a season has recently been related to sporting success, showing that the lower the number of sporting injuries, the higher the performance [16]. In particular, soft tissue injuries involving muscle, tendons, and ligaments are very common at all levels of sport [17]. The most frequent injuries are muscle (especially in the hamstring muscles [18]), ligament (i.e., anterior cruciate ligament rupture [19]), and joint (i.e., ankle sprain [8]) injuries. In fact, up to 80% of injuries generally affect the musculoskeletal tissue. For instance, deltoid muscle injuries per year are between 12 and 19% in baseball players and between 23 and 38% in swimmers [20]. In marathon runners, the incidence of training-related lower limb muscle injuries is estimated to be between 19 and 58% [21]. In tennis, about 3.49 injuries/1000 h have been reported frequently in joints (29.5%), tendinopathies (22.1%), ankle (20%), and wrist (15.8%) [22].

Generally, two stages of management can be considered in the rehabilitation process from a sport injury [23]. The first stage corresponds to the phase of immobilization, atrophy, and subsequent tissue repair. This stage can last for several days or months depending on the severity of the injury. Normally, it is a period that leads to deconditioning due to the lack of movement of the affected body section. This might evoke significant loss of muscle mass as well as functional alterations of the musculoskeletal and connective tissues [24]. Some nutritional strategies are suggested to contribute to the protection/repair of muscle tissue and modulation of the immune system by controlling catabolic and inflammation processes through the regulation of reactive oxygen species (ROS) production and catabolic pathways [17,25,26]. However, to date, the effect of these nutrients on the rehabilitation of elite athletes is unclear or even ambiguous in certain contexts [27]. The second stage corresponds to the readaptation to training and improvement of the psychological profile (i.e., emotional level) of the athlete. Some authors refer to this as the reathletization phase [28]. We have recently highlighted the compensatory neural changes (e.g., brain cortical changes) and the cognitive load that might affect recovery and relapse after a musculoskeletal injury [29]. Since early mobilization and stimulation of the affected tissue (i.e., low-intensity pulsed ultrasound, neuromuscular electric stimulation) has been shown to have a positive effect on collagen reorganization and general connective tissue repair, it is recommended to start with a controlled loading program as soon as pain or injury permits [6]. It is worth noting that medical personnel, physiotherapists, and athletic trainers should respect the natural healing process of the human body and ensure a balance between workload and rest time to avoid longer lasting tissue damage [30,31].

To facilitate the recovery of physical-related parameters, a multidisciplinary team of sports practitioners is needed to cover the energetic, nutritional, and psychological among other demands of the injured elite athletes. Our group has emphasized that adopting a systemic (integrative and multifactorial), evolutionary (intuitive), and adaptive (ever-changing based on individualization) perspective or ‘Bio-Logic approach’ [32] would enhance our understanding of the flow of information through interactions between system components and their regulatory aspects for a given phenotype and the allostatic load. Indeed, the allostatic load (as the cost a biological system must pay in order to reset physiological parameters [e.g., injury recovery] during the adaptation [33]) has been proposed as a promising and underutilized measure that might be useful to assess the spinal cord injury time course [34]. Importantly, nutrition is one of the many factors that might impact the allostatic load and, thereby, it might influence the musculoskeletal tissue overload and repair. Therefore, a special nutritional intervention throughout the rehabilitation process is warranted to ensure integral recovery while accelerating tissue regeneration [24]. In this regard, it should be noted that tissue repair is a high energy-consuming process (i.e., protein synthesis, cytoskeleton remodeling, etc.). As a result, the energetic and protein deficiency might hamper proper healing and increase the inflammatory response which would decrease the rate of tissue recovery while increase injury relapse [23]. In this sense, the aim of this systematic integrative review was to update the effective nutritional strategies that benefit the rehabilitation of musculoskeletal injuries in elite athletes.

## 2. Methods

This study employed the five stages developed by Whittemore and Knafl [35] as the established guidelines of the integrative review. This allows for the combination of past empirical or theoretical literature to provide a more comprehensive understanding of a particular phenomenon or healthcare problem, which has a greater impact to establish evidence-based recommendations. The aim was to synthesize the occurrence of literature regarding nutrition interventions for the injured athlete. Similar to previously published articles [36], the review methodology was enhanced by optimizing the stages of literature search, data evaluation, and data analysis in order to systematize the review process and improve the scientific soundness according to recommendations given by Hopia et al. [37] and the PRISMA in Exercise, Rehabilitation, Sport Medicine and Sports Science (PERSiST) guidelines [38]. The protocol of this review was published and freely accessible at Figshare to avoid unnecessary duplication (DOI: 10.6084/m9.figshare.21399696).

### 2.1. Eligibility Criteria

The inclusion criteria for this review were as follows: (1) Empirical or theoretical articles (quantitative, qualitative, mixed method studies, and systematic reviews) that assessed or included elite/high-performance male and female athletes over 18 years of age. Only review articles that evaluated the use of nutrients in the rehabilitation phase after a musculoskeletal sports injury were reported or discussed; (2) studies were published between 2012 and 2022; (3) articles were written in the English and Spanish language; (4) available in full text; and (5) focused solely on the assessment of nutritional (energy intake, macronutrient distribution, micronutrients, etc.) or supplementation strategies during the rehabilitation process in injured athletes. On the other hand, the exclusion criteria consisted of articles that: (1) Included children, older adults, physically active people, amateur or recreational population and non-conventional athletes; (2) commentaries, dissertations, theses, editorials, letters to the editor, and books; (3) interventions where the dosage and timing of intake of nutrients and sports supplements were not specified; and (4) articles that did not analyze the relationship between nutrition and musculoskeletal sports injuries (e.g., concussions).

### 2.2. Information Sources

The following academic databases were selected to examine the literature: PubMed/Medline, Scopus, PEDro, and Google Scholar.

### 2.3. Search Strategy

The patient, intervention, comparison, and outcome (PICO) strategy was utilized for structuring the research question: P (athletes aged >18 years old) I (nutritional intervention) C (placebo, or non-exposed control group [pre-post]) O (musculoskeletal recovery- or rehabilitation-related outcomes) [39]. The authors followed the identical string in searching the databases to ensure consistency with the data search, as follows: (i) Pubmed/MedLine, (Nutrition OR supplementation) AND sports AND inju*, and “sports injuries” OR “athletic injuries” OR “sport injury rehabilitation” AND (nutrition OR dietary supplements); (ii) Scopus, “sports injuries” OR “athletic injuries” OR “sport injury rehabilitation” AND (nutrition OR dietary supplements). In addition, further papers were hand searched (e.g., snowballing) in the databases. The data search in PEDro and Google Scholar was performed using free language terms, such as nutrition, supplementation, and musculoskeletal injuries.

### 2.4. Selection Process

After executing Boolean algorithms, filters were used in the different databases to select potentially eligible articles. Four authors independently evaluated the databases for articles that met the inclusion criteria (J.E.G-V., M.A.C-G., E.J.R-A., and D.A.B.). Discrepancies were identified and resolved through discussion (with a fourth author where necessary). Those publications that met all the requirements went on to the next phase of data analysis and synthesis. The database search took place during June and October 2022 to capture relevant articles for the review, although an updated search was conducted prior to manuscript submission.

### 2.5. Data Collection Process and Items

A table to synthesize results and findings was built with the following data: (i) General information on the study (title, author, year, and type of study); (ii) description of the study population; (iii) study aim and methodology; (iv) characteristics of the nutritional and/or supplementation strategy (timing and dosage); and (v) main findings of the study. 

### 2.6. Study Risk of Bias Assessment

Risk of bias assessment for randomized clinical studies was performed using the Cochrane RoB 2.0 tool (RoB2 Development Group, University of Bristol, Bristol, UK) [40]. Five bias domains (randomization process, deviations from intended interventions, missing outcome data, outcome measurement, and selection of the reported outcomes) were evaluated [41]. The overall assessment of the risk of bias for each outcome was presented as: ‘Low risk’, ‘some concerns’, or ‘high risk’ of bias. We used the AMSTAR 2.0 checklist in order to assess the methodological quality of the selected review articles [42]. The 16 items presented to determine the classification of the systematic review as ‘reliable’ or ‘not very valid’ were considered [43].

## 3. Results

### 3.1. Study Selection

**Figure 1:**
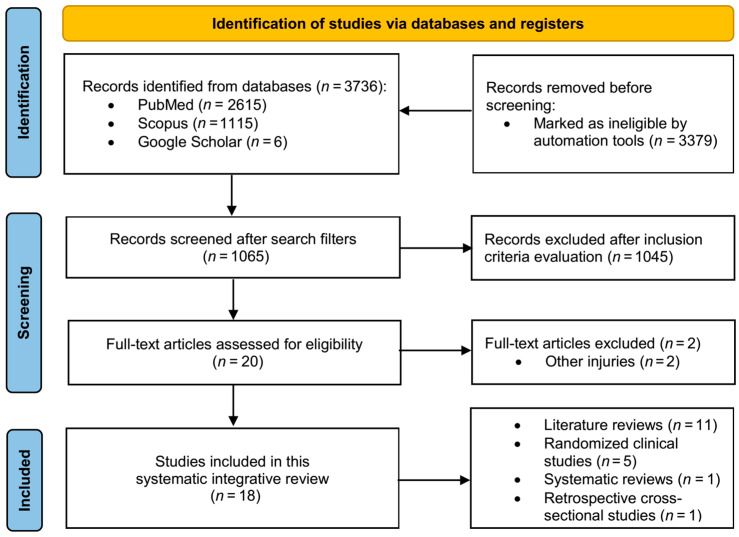
PRISMA flow diagram.

After running the search algorithms with Boolean operators and free language terms, 3736 references were obtained. Filtering by date, type of article, language, and availability of full text resulted in 1065 potentially eligible studies. It should be noted that +100 clinical trials were published between 1992 and 2012. However, after screening the abstracts and full texts of these articles and analyzing strict compliance with inclusion criteria, 1045 articles were excluded. A total of 18 studies met the requirements of this integrative systematic review (Price Index: 66.6%). Figure 1 shows a flow diagram of the literature search.

### 3.2. Risk of Bias within Studies

**Figure 2:**
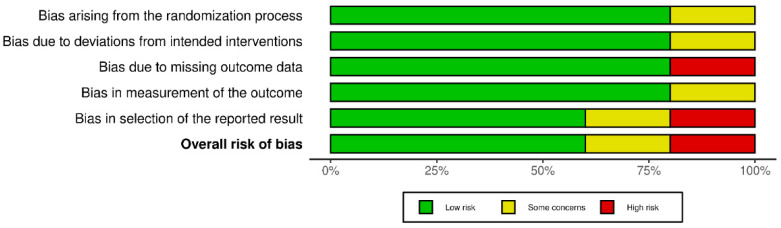
Risk of bias summary for included studies. Weighed bar-chart of the distribution of risk-of-bias judgments. These graphics were obtained using the ‘robvis’ package within the R statistical computing environment.

Compared to review articles, fewer clinical trials have been carried out in the last 10 years. The methodological quality of the five randomized clinical trials included in this integrative systematic review is shown in Figure 2.

Similarly, the methodological quality of the twelve reviews included in this systematic integrative evaluation of the literature was performed with the AMSTAR 2.0 tool (Table 1). In general, a classification of low quality (high risk of bias) was found in the selected review articles. Therefore, a lack of reproducibility and replicability of the reviews performed on this topic is notable to date. Only one retrospective cross-sectional study that evaluated injured Australian and international athletes was included [44].

### 3.3. Results of Individual Studies

Table 2 presents a synthesis of the scientific evidence on the nutritional strategies that have been evaluated during the rehabilitation of musculoskeletal injuries in elite athletes.

## 4. Discussion

Sports injuries represent a major economic expense with more than USD 9 billion spent annually on injury recovery and rehabilitation in young adult athletes (17 to 44 years old) [48]. Traditionally, rehabilitation management of sports injuries has been approached from the area of physiotherapy and sports medicine by means of mechanical activities (such as local cold, heat, massage, extracorporeal shock waves, isometric exercises, etc.), anti-inflammatory drugs (paracetamol, non-steroidal anti-inflammatory drugs [NSAIDs], ibuprofen, diclofenac, betamethasone, and muscle relaxants) and surgical interventions [60]. However, sports nutrition and supplementation play an important role as non-pharmacological strategies during the different stages of inflammation and healing of musculoskeletal injuries in the athlete. Considering the volume of evidence analyzed in this study, nutritional strategies have been shown to be effective in optimizing the management of inflammation, injury-generated oxidative stress and, in general, the process of musculoskeletal tissue repair [48,61]. In general, study findings agree on the importance of monitoring energy availability and dietary protein intake since they play a fundamental role in the recovery process during sports injury. Nevertheless, in light of the current evidence it is not possible to draw definitive conclusions and recommend supplementation with other nutrients (e.g., collagen, Omega-3 fatty acids, creatine, vitamin D, β-hydroxy-β-methylbutyrate [HMB], glucosamine, probiotics, and other micronutrients [Ca and Zn]) given the few number of controlled clinical trials. This has been frequently stated in recent review articles [50,62]. The following sections of this integrative systematic review describe in detail the advances of the last years regarding nutritional strategies that deserve attention and may be applied during the rehabilitation process of musculoskeletal injuries in the elite athlete.

### 4.1. Energy Availability

Nutrition practitioners should provide supportive supervision of energy intake by the injured athlete (as close as possible to energy balance or even slightly superior). Even though the period of injury or immobilization may entail a decrease in physical activity, it has been reported that energy expenditure may be higher (≈20%) during the early phases, especially in severe injuries [23,48]. It should be highlighted that the process of muscle protein synthesis (MPS) is high energy-demanding, ranging from ≈485 kcal/day in a muscular young man to ≈120 kcal/day in an active elderly woman [24]. Some studies have concluded that an energy deficit of 20% can lead to a decrease in MPS of about 19% [30]. Moreover, genome and cytoskeleton remodeling are another energetically costly function of any cell [63,64]. Therefore, drastic decreases in energy intake can accelerate muscle mass loss by decreasing MPS and facilitating muscle protein breakdown (MPB), which hinders the rehabilitation process. Complementarily, it should be considered that excess energy results in an increase in adipose tissue and systemic inflammation, which aggravates the loss of muscle mass [31]. This higher energy cost has been recently referred to as the ‘allostasis and stress-induced energy expenditure’ and, therefore, is part of the allostatic overload that takes place during the musculoskeletal injury [65].

Energy availability (EA) is defined as the amount of energy available to maintain metabolic function after subtracting the exercise energy expenditure (EEE) from energy intake (EI) [66]. The assessment of EA is used as a diagnostic tool for the management of relative energy deficiency in sport (RED-S) and it is expressed as: EA = EI − EEE/FFM [67,68,69]. There is evidence that to have a healthy physiological function in athletes engaged in preparation or competition activities, it is determined that EA should be ≈45 kcal/kg FFM per day [69]. An insufficient energy intake might evoke in EA below the previous recommended value which is known as low energy availability (LEA). This can seriously compromise the body functions required to maintain optimal health and physical performance. In fact, health alterations may occur after only 5 days of LEA in women [70]. It is worth noting that most of the included articles in this integrative review agree with this recommendation to sports practitioners: Avoid EA values <30 kcal/kg FFM/day [10,13,44,46,48,49,58]. LEA may cause strong alterations in the endocrine system [66] which encompass disruption of the hypothalamic-pituitary-gonadal axis, thyroid, appetite-regulating and sex hormones (testosterone and progesterone) [71], decreased insulin and insulin-like growth factor 1 (IGF-1), increased growth hormone (GH) resistance and cortisol elevations. Furthermore, profound alterations have been reported on bone health [72], metabolic profile, and cardiovascular, gastrointestinal, immune, and psychological function in addition to eating disorders and decreased athletic performance [67,69,73]. This low energy scenario not only increases the risk of musculoskeletal injuries, but also hampers the natural healing process of rehabilitation. LEA prevention during injury recovery in elite athletes requires a commitment between the athlete and sports practitioners [69] with special attention to the education component to raise awareness of unwanted effects [74]. In this sense, tools that have not yet been validated have been designed to help in the prevention and early detection of LEA for the prevention of long-term sequelae [75].

### 4.2. Loss of Muscle Mass and Protein Intake

Whereas an increase in mechanical stress stimulates an anabolic response with consequent muscle hypertrophy, situations of immobilization or disuse generate the opposite effect by increasing anabolic resistance resulting in muscle atrophy [76]. It has been estimated that during immobilization ≈0.5–0.6% of muscle mass is lost per day. Therefore, this worrisome loss of muscle mass is accompanied by an even greater loss of strength [1]. As a result, not only muscle structural atrophy, but also neuromuscular degeneration may occur [2]. These periods of immobilization are also associated with a loss of bone mineral density in most parts of the body [3], which increases the risk of fractures especially in the elderly or in subjects that due to nutritional deficiencies and high loads generate significant bone demineralization (cases that may occur in athletes with eating disorders or who undergo very strict weight loss regimens with high training loads). In addition, the cardiovascular and cardiorespiratory systems are largely affected by this immobilization condition. Of note, a daily loss of 0.99 and 1.6% in maximal oxygen consumption (VO_2max_) and cardiac output, respectively, after only 2 weeks of bed rest have been reported [4]. Interestingly, the longitudinal Dallas Bedrest and Training study showed that VO_2max_ decreased more after 3 weeks of bedrest than during 30 years of aging [5]. It is important to point out that due to the loss of muscle mass and physical inactivity, periods of disuse also induce an alteration of the metabolic state, favoring an increase in insulin resistance [6]. 

In relation to the loss of muscle mass, it is known that the net protein balance is the difference between MPS and MPB [11,16]. Therefore, a MPB greater than MPS would induce a decrease in muscle mass, especially if a LEA is present. Different studies have shown that in the first days of injury, the MPB is transiently elevated [7,8,14]. This transient increase could be the cause of the high loss of muscle mass that occurs in the first days after injury. However, it is currently considered that the energy-related decrease in MPS is the main cause of the disuse in muscle atrophy observed for periods of more than 2 weeks [14]. Another factor contributing to the loss of muscle mass during the immobilization period is the previously mentioned anabolic resistance, which is defined as the inability of an anabolic stimulus (e.g., protein, hormonal stimulation, and/or muscle tension) to stimulate SPM caused by aging, periods of inactivity, or during critical illnesses [15]. In this regard, Wall et al. [14] showed how SPM, in response to 20 g protein intake, was ≈31% lower after immobilization which indicates a decrease in tissue sensitivity to amino acids. Moreover, this might be explained by the energy crisis and intrinsic restrictions in the injured tissue which disrupt both extra- and intracellular energy production pathways and cytoskeleton organization during the allodynamic response [65]. 

Although the cellular mechanisms inherent in the process of immobilization-induced atrophy are unclear, the following are postulated: (i) The reduction in myogenic capacity (i.e., decreased satellite cell content and functionality) [1]; (ii) the mitochondrial dysfunction with consequent increase in free radicals and increased inflammatory response [3]; and (iii) an imbalance in the protein synthesis/degradation balance due to an inhibition of anabolic signaling pathways (i.e., PI3K/PDK/PKB/mTORC1) and activation of proteolytic pathways (i.e., ubiquitin-proteosome, calpain and caspase system, cellular autophagy, etc.) [11]. Therefore, periods of absence of stimulation have important consequences at a multisystemic level, as evidenced in older adults. Reducing daily physical activity generates considerable changes, such as 14% reduction in energy expenditure, lower insulin sensitivity (−43%), decreased lipid metabolism, increased visceral fat, decreased cardiorespiratory capacity, increased inflammatory markers, and a decrease in postprandial protein synthesis (26%) and leg muscle mass (−3.9%) [16]. Other studies conducted during controlled periods of immobilization in young subjects have shown a reduction in the expression and concentration of the glucose transporter in muscle (SLC2A4, also known as GLUT-4), resulting in reduced glucose tolerance [19].

Considering all of the above, a fundamental nutrient for injury recovery in athletes is dietary protein [77]. Inadequate protein intake will lead to increased loss of muscle mass, decreased tissue repair and healing, inflammation and impaired healing, all of which are MPS-dependent processes. At the same time, the anabolic resistance produced during the immobilization process generates an increase in the requirements of this macronutrient. In this regard, in vitro research [76] has demonstrated the action of amino acids on satellite cell dynamics, revealing that protein supplementation appears to accelerate satellite cell responses after acute muscle damage. This may be important in muscle remodeling and injury recovery processes. On the other hand, studies in humans suggest that dietary protein may have an important effect on the activity of satellite cells after exercise in untrained people, where there is greater muscle damage after exercise [76]. Additionally, as a protective measure, it has been shown that the inclusion of protein intake prior to sleep may be another strategy to improve muscle mass retention during periods of injury, as has been shown in studies with energy restriction [78]. Regarding protein intake, there are three fundamental factors: The quantity, the quality of the protein (source), and the time and frequency of consumption, all considering the total energy intake of the individual. In energy-restricted feeding programs, it has been observed that a higher protein intake leads to a lower loss of muscle mass. For example, Mettler et al. [79] conducted a 2-week study where participants were subjected to a 40% energy restriction and divided into two groups: One group was given 1 g of protein per kg/day, while the other was given 2.3 g/kg/day. The subjects in the group that consumed less protein reported a muscle mass loss of 1.6 kg compared to 0.3 kg in the group that consumed a higher protein diet. Most of the reviewed articles included in this integrative review recommend a high protein diet (from 1.6 to 3 g/kg/day) with 20–30 g of leucine-rich protein (≈3 g) per meal throughout the day (including pre-sleep intake). This dosage per meal (0.3 g of protein per kg per meal) has been shown to be effective in increasing MPS in young [19] and older adults [80]. It needs to be noted that a uniform distribution of proteins over a 24-h period is more favorable than when quantities are distributed unevenly [81]. Indeed, Mamerow et al. [82] showed that a homogeneous distribution of protein consumption increases MPS by more than 25% compared to a distribution where protein is mainly concentrated in the evening meal [82]. Based on this physiological response, it seems that the injured athlete will also benefit from eating 4–6 protein meals throughout the day to prevent loss of muscle mass [83]. Pre-sleep casein protein ingestion seems to be an effective strategy to boost the muscle adaptive response during a resistance exercise program [84], but more research is needed in exercise rehabilitation programs.

### 4.3. Tissue Repair and Inflammation

#### 4.3.1. Creatine Monohydrate

The most widely studied and safest nutritional supplement is creatine, especially in the form of creatine monohydrate (CrM) [85]. Its administration results in increases in the total musculoskeletal creatine pool by around 25% (up to ≈37% if accompanied with physical exercise) [86] which benefits the athlete’s recovery time and improves athletic performance (increased strength, muscle mass, and power) [87]. It has been reported that the increase in muscle creatine after CrM supplementation might optimize the function of the creatine kinase/phosphocreatine system and subsequently benefit energy- and mechanical-dependent processes in different tissues [88,89]. 

Potential effects of CrM consumption as a therapeutic nutritional agent in clinical conditions have been suggested for some chronic and traumatic diseases (acute injuries, spinal cord injury, postoperative orthopedic recovery, muscular dystrophy, immobility, and atrophy due to muscle disuse, among others) [90]. Furthermore, CrM supplementation could help in maintaining or improving clinical outcomes by improving physiological adaptations during rehabilitation processes in patients with substantially reduced skeletal muscle contractile capacity [91], as in the case of sports injuries. Periods of extreme inactivity, such as periods of immobilization, have shown not only a loss of muscle mass and strength, but also a 24% decrease in muscle creatine stores [92]. Consequently, maintaining or increasing muscle creatine levels during periods of inactivity or recovery from injury may offer benefits [93]. Indeed, several studies have evaluated the potential effects of CrM supplementation during periods of immobilization [94] revealing: (i) Maintenance of muscle mass or cross-sectional area, muscle strength, and endurance; (ii) maintenance or increase in total muscle creatine concentration; (iii) maintenance of GLUT-4 concentration [95]; (iv) increased muscle glycogen; and (v) increased expression of growth factors (IGF-1) and myogenic regulatory factors [88,93]. However, it is difficult to draw definitive conclusions due to heterogeneity in study designs (e.g., duration, immobilized limb, experience level of participants, etc.). For example, Johnson et al. [96] showed how CrM supplementation reduced muscle mass loss in immobilized arms; however, another study failed to demonstrate the same effect in lower limbs after a short-term protocol of CrM supplementation [97]. A clinical intervention during a period of 10 weeks of rehabilitation, showed that CrM intake favors the increase in muscle mass after immobilization-induced loss [98]. In particular, it seems that CrM supplementation may be effective, not over short but longer periods of time, although the overall impact on reducing muscle loss is inconclusive [83]. Recently, a randomized controlled clinical trial conducted by Juhasz et al. (2018) [57] concluded that CrM supplementation (20 g for 5 days followed by 5 g for the rest of the study) combined with therapeutic strategy effectively supports the rehabilitation of tendon overuse injury of adolescent fin swimmers. A recent systematic review that evaluated pre- and post-surgical nutrition for preservation of muscle mass, strength, and functionality also concluded that CrM supplementation merits consideration in the general population [99]. In agreement with the collective body of evidence reviewed in this systematic integrative review, we adhere to this recommendation considering the very good safety profile of CrM at doses of 0.1 g/kg/day. Notwithstanding, the few clinical studies on the effects of CrM within elite athletic population warrant more research as concluded by Mistry et al. (2022) in a recent systematic review [100].

#### 4.3.2. Omega-3 Fatty Acids

Inflammation is part of the natural tissue recovery process; therefore, a drastic reduction (using drugs or other substances) or an excess of acute inflammation could result in an inadequate physiological response and lead to a suboptimal recovery. Under normal conditions, muscle injuries generate a complex and coordinated inflammatory response that is characterized by: (i) The activation of both endothelial cells in the vessels supplying the muscle and cells residing in the muscle tissue, such as satellite cells, fibroblasts, and leukocytes (macrophages, CD8+ T lymphocytes, mast cells, eosinophils and, later, regulatory CD4^+^CD25^+^FOXP3^+^ T lymphocytes; and (ii) the recruitment and subsequent infiltration into the injured muscle of various leukocytes, especially neutrophils and monocytes (which differentiate into macrophages) [101,102,103,104].

This acute response initially generates pain, swelling, and loss of function [101]. Therefore, the use of anti-inflammatory strategies that include long-chain fatty acids of the omega-3 family might be useful for short periods of time. Eicosapentaenoic acid (EPA) and docosahexaenoic acid (DHA) [105,106,107] have been shown to decrease the concentrations of some inflammatory markers, pain intensity, and the use of NSAIDs in some inflammatory diseases [105]. Moreover, other molecules derived from both EPA (e.g., resolvins E) and DHA (e.g., resolvins D, maresins, and protectins) have been shown to induce the resolution of inflammation [108]. Finally, these molecules as well as EPA and DHA are known to directly or indirectly affect transcription factors that regulate the expression of genes encoding inflammatory proteins (e.g., cytokines, chemokines, enzymes, and adhesion molecules) [13]. 

Clinical trials on the role of omega-3 fatty acids in sports-induced inflammation have focused primarily on exercise-induced muscle damage and its respective consequences (i.e., soreness, muscle swelling, loss of strength, and decreased range of motion) [106]. However, the evidence is not consistent and does not allow for extracting clear recommendations regarding amounts and timing due to conflicting results and methodological limitations [22,109]. In fact, in relation to its role in sports injuries, most research has focused on traumatic brain injury and suggests that DHA may have positive effects [22,25]. On the other hand, it has been described that the acute inflammation generated by a muscle injury is a necessary physiological response and, therefore, its reduction or blockage compromises the process of repair and regeneration of muscle tissue [101,102,103]. As a result, it has been suggested that a high intake of EPA and DHA could have negative effects in the first days post-injury [110]. 

On the other hand, it is known that arachidonic acid not only originates from eicosanoids that participate in the initial inflammatory response, but also gives rise to lipoxins that along with resolvins, protectins, and maresins (derived from EPA and DHA) act as mediators of the resolution of inflammation [10,49]. This suggests that perhaps the ratio of arachidonic acid to the sum [EPA+DHA] is as or more relevant than total omega-3 intake. However, arachidonic acid is not the most abundant omega-6 fatty acid in typical diets of industrialized countries [10], but is a linoleic acid. This latter function reduces the conversion of α-linolenic acid of the omega-3 family to EPA and DHA, thus competing with cell membrane phospholipids [49,61]. Therefore, considering the lack of clear evidence to establish guidelines for the intake of these fatty acids in athletes, when the aim is to prevent or assist in the treatment of muscle injury, it is considered more prudent to recommend that the athlete’s diet has a low omega-6/omega-3 ratio.

#### 4.3.3. Collagen Peptides and Specific Gelatin Products 

Tendon injuries are quite frequent in athletes, and their origin is multifactorial. One randomized, double-blind, crossover study showed that the combination of jumping exercise together with gelatin and vitamin C supplementation (15 g gelatin + 50 mg vitamin C) increased in vitro collagen production and a two-fold increase in amino terminal propeptide of type I collagen in blood, which is indicative of increased collagen synthesis [111]. This suggests that the inclusion of collagen peptides and specific-gelatin products in combination with an intermittent exercise program may enhance collagen synthesis, which could play a beneficial role in injury prevention and tissue repair. In this sense, research has shown that supplementation with hydrolyzed collagen (≈10 g per day) can increase cartilage thickness in patients with osteoarthritis [112] and decrease knee pain in athletes [113]. Indeed, the recent systematic review performed by Kahtri et al. (2021) [47] concluded that ‘collagen peptides and specific gelatin products have strong evidence in improving joint pain and functionality (especially at doses of 15 g/day)’. The data presented suggest a benefit of gelatin along with vitamin C and/or hydrolyzed collagen supplements; therefore, it is expected that future clinical studies need to confirm this recommendation in injury-related specific conditions [93].

#### 4.3.4. HMB

β-hydroxy-β-methylbutyrate (HMB) is a leucine-derived metabolite marketed as a supplement to increase MPS and decrease MPB [93]. Different mechanisms have been proposed to justify its anti-catabolic action including activation of the PI3K/Akt/PDK/IGF-1/mTORC1 signaling pathway [114]. However, the same benefit can be obtained with the ingestion of leucine or whey protein [115]. The effects of this substance on muscle mass gain and muscle damage are unclear in trained athletes [116,117]. In fact, there is controversy in the literature due to the different study designs, the lack of transparency, and even the possible conflict of interest in some studies [93]. Despite the above, HMB is recommended by some of the reviews evaluated [45,52], which highlight that it can be useful in rehabilitation characterized by periods of extreme inactivity. For instance, Deutz et al. [118] showed improved lean mass preservation in older adults ingesting HMB during 10 days of bed rest. However, only one pilot study with eight federated athletes diagnosed with patellar tendinopathy has shown positive effects with doses of ≈3 g/day before the exercise rehabilitation program [59]. Based on the accumulated evidence, unlike other supplements, such as CrM, HMB cannot be confidently recommended to injured athletes since the effects may not be more effective than following current protein intake recommendations [93,107]. 

#### 4.3.5. Vitamin D

There is a growing body of literature highlighting the importance of vitamin D, beyond its classically described effects on phosphorus and calcium metabolism in bone [119,120,121]. Importantly, a high prevalence of vitamin D deficiency in the athlete population has been highlighted and, given the important role it plays in the adaptive processes to intense exercise [93], it is necessary to monitor and maintain adequate levels in preparation and competition phases. Regarding the relationship between vitamin D and muscle performance, it is known that vitamin D binds to vitamin D receptors in muscle tissue to regulate gene expression in muscle fibers (especially type II). A study in 22 judo athletes with vitamin D deficiency showed an improvement in isokinetic dynamometry in quadriceps and hamstring strength tests with daily intake of 150,000 IU of vitamin D3 for 8 days [122].

Regarding athletic injuries, low serum 25-hydroxyvitamin D levels have been associated with increased risk of stress fracture by 3.6 times in Finnish military recruits [123]. Similarly, vitamin D insufficiency results in 1.86 times the risk of lower extremity muscle strain and 3.86 times the risk of hamstring injury in athletes in the National Football League [124]. It should be noted that vitamin D insufficiency is established at <80 nmol/L while deficiency is at <50 nmol/L [44]. It has also been documented that supplementation with 800 IU of vitamin D3 in addition to 2 g/day of calcium reduced stress fractures in female recruits by 20% [125]. In a study of National Football League players, it was found that those with at least one muscle injury had significantly lower vitamin D levels than those with no injuries during the season [126]. Complementarily, serum 25-hydroxyvitamin D levels after vitamin D supplementation not only increases but has a significantly negative correlation with selected biomarkers of skeletal muscle damage and post-exercise levels of pro-inflammatory cytokines [127]. 

In addition to the association with risk of injury, vitamin D may also influence recovery after some types of surgery. Barker et al. [128] observed that subjects with low vitamin D levels had delayed strength recovery after anterior cruciate ligament surgery. Since low levels are associated with risk of injury (e.g., stress fractures, muscle injuries, and upper respiratory tract infections) [129], vitamin D supplementation in athletes with low serum 25-hydroxyvitamin D concentrations would be indicated as an adjuvant strategy to decrease the rate of injury. In athletes with spinal cord injury, benefits of oral vitamin D supplementation (6000 IU/day) for 12 weeks to correct deficiencies have been reported in Swiss para-athletes [56]. Despite the above-mentioned research, contradictory results have been found that do not allow for a clear conclusion due, to a large extent, to the diversity in the designs of these investigations (e.g., differences in baseline 25-hydroxyvitamin D levels, supplementation protocol, number of participants, etc.) [130]. Therefore, more studies with solid intervention designs are needed to evaluate vitamin D supplementation in the recovery process after a sports injury [131].

### 4.4. Future Directions

In general, nutritional recommendations for rehabilitation and return to competition are similar to those made for muscle gain [18], which may be due to the increased need for energy and higher protein intake in order to avoid loss of muscle mass [19]. However, despite current knowledge in sports nutrition, there is insufficient clinical information on the use of certain nutrients in the injured athlete [17]. Importantly, the effects on improving musculoskeletal and tendon/ligament tissue function in the injured athlete requires further research. For this reason, it is important to develop future studies to evaluate the clinical effects of these nutrients during the injury rehabilitation program in the athletic population.

One of the factors contributing to muscle atrophy induced by physical inactivity or immobilization is ROS production [132]. This seems to be due to its interference with the MPS process by hindering translation initiation [133]. In addition, it has been observed that these ROS can activate different proteolytic systems, such as autophagy, calpain, or the ubiquitin proteasome system, which increases proteolysis and susceptibility to amino acid oxidation [132]. The use of antioxidants can be effective in decreasing immobilization-induced muscle atrophy, albeit, this is currently a controversial issue. In recent years, evidence is accumulating on the role of ROS as cellular signaling and their involvement in exercise adaptation processes (e.g., mitochondrial dynamics/biogenesis, insulin sensitivity, muscle hypertrophy, antioxidant enzyme expression, etc.) [134,135,136,137]. Therefore, it has been reported that high doses of antioxidant supplements can block the exercise-induced adaptive response of muscle tissue [138]. For example, Barker et al. [139] showed how vitamin C and vitamin E intake can negatively influence the recovery of muscle function after knee surgery, although in this study, adequate prior vitamin C status was correlated with better muscle function. These data suggest that a correct antioxidant status is necessary to maintain physiological ROS ranges and, therefore, permissive to all adaptive processes. An interesting point to note is that there is no data that high intakes of fruits and vegetables (sources of antioxidants) attenuate adaptations to exercise; therefore, it would be appropriate to recommend that athletes consume a quality diet and avoid mega doses of antioxidant supplements and micronutrients [140]. Recommendations for injured athletes comprise the intake of antioxidants and micronutrients through a varied and balanced diet, rich in protein, fiber, fruits, and vegetables, which might support the maintenance of antioxidant status. Future intervention studies should provide more evidence on the need for antioxidant supplementation on recovery after injury, considering aspects, such as dose and type of antioxidant used. For example, curcumin and derivates (curcuminoids) are mentioned in the UEFA expert group statement as a potential strategy to combat the acute inflammatory process during the injury rehabilitation program; however, they also highlight the fact that it requires corroboration in relevant human studies to recommend its use [58]. Doses used at the time of supplementation are generally safe up to about 5 g/day [93] although some side effects, such as nausea, diarrhea, headache, and yellow stools have been reported [141]. Finally, in view of its possible role as a neuroprotective agent with analgesic effects [142], it has been suggested that melatonin supplementation before physical exercise could be a strategy in the rehabilitation of spinal cord injuries [143]. Melatonin has good tolerability after short-term use and, thereby, is a good candidate molecule to perform clinical trials in injured athletes.

## 5. Limitations and Strengths

This review should be read in light of various limitations/strengths. First, it focused on outcomes related to the treatment or intervention of serious and non-serious injuries reported within each study. While partial generalizability may take place, it is worth noting that we did not fully cover prevention of injuries, injury-associated risk factors, nor other types of injuries (e.g., traumatic brain injury). Even though it is beyond the scope of this work, sports practitioners are encouraged to facilitate nutritional post-exercise recovery [84] and follow injury prevention programs based on international consensus [144] along with the findings of this comprehensive review of the literature. It is the first time that scientific evidence on this topic is analyzed under a systematized methodology that included quality and risk of bias assessment. Finally, conclusions and recommendations given in this systematic integrative review should be discussed carefully in other populations (e.g., physically active individuals) in contrast to athletes.

## 6. Conclusions

Nutritional strategies that would most likely benefit the rehabilitation process in injured athletes include energy availability, and high protein and carbohydrate diets. Importantly, supportive supervision should be provided to avoid LEA. Considering the current evidence, it is not possible to draw definitive conclusions on supplementation with other nutrients, such as collagen, Omega-3 fatty acids, creatine, vitamin D, HMB, glucosamine, and other micronutrients given the few numbers of controlled clinical trials. After analyzing the full body of evidence, study findings agree on the importance of monitoring energy availability and the high protein intake; however, there is a notable lack of clinical research evaluating nutritional supplements in injured athletes. It should be noted that a higher number of literature review articles has been published in the last 10 years compared to clinical studies. While a low-to-moderate risk of bias was detected in the selected clinical trials, a low quality and high risk of bias were common among the review articles (mainly narrative). Therefore, researchers are encouraged to conduct further experimental studies evaluating the discussed nutrients and to follow international review guidelines at the time of reviewing literature to enhance quality and transparency. 

## Figures and Tables

**Table 1:** Quality assessment checklist for included review articles.

AMSTAR QUESTIONS	Papadopoulou et al. 2022 [10]	Burton et al. 2022 [45]	Turnagol et al. 2022 [46]	Khatri et al. 2021 [47]	Smith-Ryan et al. 2020 [48]	Papadopoulou et al. 2020 [49]	Close et al. 2019 [13]	Quintero et al. 2018 [50]	Kahn et al. 2015 [51]	Tipton 2015 [17]	Wall et al. 2015 [52]	Pyne et al. 2014 [53]
Did the research questions and inclusion criteria for the review include the components of PICO?	Yes	Yes	Yes	Yes	No	Yes	Yes	Yes	Yes	Yes	Yes	Yes
Did the report of the review contain an explicit statement that the review methods were established prior to the conduct of the review and did the report justify any significant deviations from the protocol?	No	Yes	No	Medium	No	No	No	Medium	No	No	No	No
Did the review authors explain their selection of the study designs for inclusion in the review?	No	Yes	No	Yes	No	No	No	Yes	No	No	No	No
Did the review authors use a comprehensive literature search strategy?	No	Medium	No	Medium	No	No	No	Medium	No	No	No	No
Did the review authors perform study selection in duplicate?	No	Yes	No	Yes	No	No	No	No	No	No	No	No
Did the review authors perform data extraction in duplicate?	No	Yes	No	Yes	No	No	No	No	No	No	No	No
Did the review authors provide a list of excluded studies and justify the exclusions?	No	Yes	No	Yes	No	No	No	Yes	No	No	No	No
Did the review authors describe the included studies in adequate detail?	No	No	No	Medium	No	No	No	No	No	No	No	No
Did the review authors use a satisfactory technique for assessing the risk of bias (RoB) in individual studies that were included in the review?	No	0	No	Yes	No	No	No	0	No	No	No	No
Did the review authors report on the sources of funding for the studies included in the review?	No	No	No	Yes	No	No	No	No	No	No	No	No
If meta-analysis was performed did the review authors use appropriate methods for statistical combination of results?	0	0	0	0	0	0	0	0	0	0	0	0
If meta-analysis was performed, did the review authors assess the potential impact of RoB in individual studies on the results of the meta-analysis or other evidence synthesis?	0	0	0	0	0	0	0	0	0	0	0	0
Did the review authors account for RoB in individual studies when interpreting/discussing the results of the review?	No	No	No	Yes	No	No	No	No	No	No	No	No
Did the review authors provide a satisfactory explanation for, and discussion of, any heterogeneity observed in the results of the review?	Yes	Yes	Yes	Yes	Yes	Yes	Yes	Yes	Yes	Yes	Yes	Yes
If they performed quantitative synthesis did the review authors carry out an adequate investigation of publication bias (small study bias) and discuss its likely impact on the results of the review?	0	0	0	0	0	0	0	0	0	0	0	0
Did the review authors report any potential sources of conflict of interest, including any funding they received for conducting the review?	Yes	Yes	Yes	Yes	Yes	Yes	Yes	Yes	Yes	Yes	Yes	Yes

**Table 2:** Synthesis of the selected articles for the integrative review.

Type of Study	Participants (M; F)	Aim	Methodology	Dosage and Timing	Main Findings	Reference
RCT/Quantitative Analysis	*n* = 45 (32 M; 13 F) high-performance athletes. Rugby (*n* = 17), Soccer (*n* = 10), Handball (*n* = 5), Judo (*n* = 4), Basketball (*n* = 3), Tennis (*n* = 2), Climbing (*n* = 1), Motocross (*n* = 1), Kitesurf (*n* = 1).	To determine the effect of a muscle 2–3-week rehabilitation program following ACL reconstruction and the influence of L-leucine supplementation on muscle strength in athletes undergoing sports reathletization.	Muscle strengthening exercises, proprioception, and running. Athletes were randomly assigned to receive L-leucine (*n* = 22) or placebo (*n* = 23). Thigh perimeter, isokinetic strength, single-leg long jump, and body fat (based on skinfolds) were measured.	330 mg of L-leucine per capsule four times per day (1.2 g leucine daily).	A muscle rehabilitation program with or without leucine favored the improvement of muscle quality. However, leucine supplementation favored the recovery of the injured muscle and a reduction of 1.28% in body fat.	Laboute et al. (2013) [54]
Narrative Review/Qualitative Analysis	74 references (from year of publication to 2015).	To summarize the physiological basis of muscle atrophy/disuse and discuss nutritional intervention strategies to limit muscle tissue loss during recovery from injury (including non-immobilization-induced disuse).	Expert view and non-structured analysis of the scientific literature.	High protein diet (1.6 to 2.5 g/kg/day) with a high leucine content (2.5–3 g). Consume 4–6 meals daily with 20–35 g per meal. 1.5 g of HMB two times per day. 4 g of Ω3 per day. 20 g of CrM per day (high or loading dose).	Specific nutritional compounds, such as Ω3, high protein diet (including leucine), CrM, and HMB may assist in maintaining muscle protein synthesis rates during a period of injury.	Wall et al. (2015) [52]
RCT/Quantitative Analysis	*n* = 30 (30 M; 0 F) Athletes who underwent arthroscopic ACL reconstruction (73% were soccer players).	To examine the effectiveness of glucosamine sulfate administration on the rehabilitation outcomes of ACL reconstructed male athletes.	Athletes were assigned to receive glucosamine (*n* = 15) or placebo (*n* = 15) during 8 weeks. Knee pain (VAS), functional status, and isokinetic strength were measured.	1000 mg of glucosamine sulfate per day for 8 weeks.	Glucosamine sulfate supplementation did not positively affect the rehabilitation outcomes.	Eraslan and Ulkar (2015) [55]
Narrative Review/Qualitative Analysis	74 references (from year of publication to 2015).	To translate the knowledge regarding the role of vitamin D in athletic injuries to sports physical therapy practice.	Expert view and non-structured analysis of the scientific literature.	4000 IU of Vitamin D per day or 50,000 IU per week for 8 weeks (to correct deficiency during rehabilitation).	Athletes with musculoskeletal injuries have significantly lower vitamin D levels relative to athletes without injuries. Treatment of vitamin D deficiency would lead to a decrease in the recurrence of musculoskeletal injuries.	Kahn et al. (2015) [51]
Narrative Review/Qualitative Analysis	136 references (from year of publication to 2015).	To examine and update the evidence for nutritional strategies to support the enhancement of recovery and return to training and competition (focus on the first stage of injury, i.e., wound healing and reduced activity or immobilization).	Expert view and non-structured analysis of the scientific literature.	High protein diet (2 to 2.5 g/kg/day). 10–20 g of EAA two times per day. Fish oil (Ω3). CrM. Micronutrients (vitamin A, vitamin C, vitamin D, Zn, etc.).	The best recommendation would be to adopt a ‘first, do no harm’ approach. The basis of nutritional strategy for an injured athlete should be a well-balanced diet based on whole foods from nature that are minimally processed.	Tipton (2015) [17]
RCT/Quantitative Analysis	*n* = 21 (21 M; 0 F) Swiss elite wheelchair indoor athletes with a spinal cord injury. Wheelchair rugby (*n* = 15), basketball (*n* = 4), or table tennis (*n* = 2).	To investigate the effect of vitamin D supplementation on muscle strength and performance in indoor wheelchair athletes.	Athletes received vitamin D for 12 weeks after detecting insufficiency at baseline. Muscle strength, power, and the extremity function and symptoms (DASH questionnaire) were measured.	6000 IU of vitamin D3 (cholecalciferol) daily over 12 weeks.	Twelve weeks of vitamin D3 supplementation appears to be sufficient to reach an optimal vitamin D status in indoor wheelchair athletes. The real effect of vitamin D supplementation on upper body exercise performance in athletes with a spinal cord injury still remains unclear.	Flueck et al. (2016) [56]
Narrative Review/Qualitative Analysis	89 references (from year of publication to 2019).	To identify nutritional strategies to assist with the most common injuries and consideration of the change in energy requirements during the injury period.	Expert view and non-structured analysis of the scientific literature.	Avoid chronic low energy availability (<30 kcal per kg of FFM per day). High protein diet (2.3 g/kg/day). 10–20 g of EAA two times per day. 5 g of Ω3 per day (2 weeks). 20 g of CrM per day (divided in four doses). 2000–4000 UI of vitamin D per day. ~1300 mg of Ca per day.	The nutritional strategies discussed in this review can be implemented to decrease risk, marked loss of muscle mass due to disuse, and recovery time in the injured athlete. Supportive supervision should be provided to avoid low energy availability.	Close et al. (2019) [13]
Narrative Review/Qualitative Analysis	80 references (from year of publication to 2018).	An overview of the nutritional strategies and recommendations after a muscular sports injury, emphasizing on muscle recovery.	Semi-structured literature search in PubMed, Science Direct, Scielo, Embase, and Google Scholar databases using specific search terms (MeSH and DeCS).	High protein diet (1.6 to 2.5 g/kg/day) with 20–35 g per meal (10 g of EAA). 3–5 mg of CoQ10 per day. ≥10^10^ CFU of *Lactobacillus acidophilus* or *Bifidobacterium longum*.	A high protein diet is recommended to maintain muscle mass. An adequate supply of antioxidant compounds and the use of probiotics might accelerate the muscle recovery process.	Quintero et al. (2018) [50]
RCT/Quantitative Analysis	*n* = 18 (10 M; 8 F) Injured adolescent male and female competitive fin swimmers.	To investigate the effect of creatine (Cr) supplementation on regeneration periods in tendon overuse injury rehabilitation of adolescent fin swimmers.	Athletes were assigned to receive CrM (*n* = 9) or placebo (*n* = 9) during 6 weeks as part of the conservative treatment of the tendinopathy. Segmental lean mass, ankle plantar flexion peak torque, pain intensity, and muscle damage were measured.	20 g of CrM for 5 days (loading phase) followed by 5 g daily for 37 days (maintenance phase).	The results of this study indicate that CrM supplementation combined with therapeutic strategy effectively supports the rehabilitation of tendon overuse injury of adolescent fin swimmers.	Juhasz et al. (2018) [57]
Narrative Review/Qualitative Analysis	316 references (from year of publication to 2020).	To provide a narrative synthesis of the scientific background related to selected topics (Expert Group Topic 7: Nutrition for Injury) within an elite sports setting.	Expert group statement with non-structured analysis of the scientific literature.	Ensure sufficient energy. High protein diet (1.6 g/kg/day) with 20–30 g per meal of leucine-rich protein (≥2.5 g) throughout the day including pre-sleep. Avoid deficiencies in Ca, Zn, Cu, Mn, vitamin D, and vitamin C.	Given the metabolic demand of tissue/wound recovery processes, staying as close to energy balance as possible and thus avoiding drastic reductions in energy intake, is perhaps the most crucial nutritional aspect during rehabilitation.	Collins et al. (2020) [58]
Narrative Review/Qualitative Analysis	77 references (from year of publication to 2020).	To define the proper nutrition for athletes in order to hasten their return to the sport after surgery or injury.	Expert view and non-structured analysis of the scientific literature.	Adequate energy intake. High protein diet (2 g/kg/day) with 20–30 g per meal of leucine-rich protein (≥2.5 g) every 2–4 h. 10–20 g of EAA. Fish oil (Ω3) and CrM. Avoid deficiencies in vitamins D and K.	Adequate intake of macronutrients can support anabolism in athletes. Dietary protocols should consider doses, timing, rehabilitation time, type, and quality of nutrients, as well as the type of injury, and the injured body part.	Papadopoulou et al. (2020) [49]
Narrative Review/Qualitative Analysis	106 references (from year of publication to 2020).	To provide an evidence-based, practical guide for athletes with injuries treated surgically or conservatively, along with healing and rehabilitation considerations.	Expert view and non-structured analysis of the scientific literature.	High protein diet (at least 1.6 and closer to 2–3 g/kg/day) of leucine-rich protein (≈3 g) per serving. 20 g of CrM per day (divided in four doses) for 5 days and then 3–5 g daily. EAA ingestion immediately before surgery or therapy. ≥10^10^ CFU of *Lactobacillus acidophilus* or *Bifidobacterium longum*.	The athlete’s energy requirements should be identified to avoid energy deficit. Higher protein intakes, with special attention to evenly distributed consumption throughout the day, will minimize loss of muscle mass and strength during times of immobilization.	Smith-Ryan et al. (2020) [48]
Systematic Review/Qualitative Analysis	48 references (from year of publication to 2020).	To evaluate the effect of COL and exercise on joint function and athletic recovery.	Structured literature search in PubMed, Web of Science, and CINAHL. Fifteen references met the inclusion criteria.	5–15 g of COL at least 1 h prior to exercise for over 3 months.	Strong evidence of COL use in improving joint pain and functionality (15 g/day may be a more effective dose).	Khatri et al. (2021) [47]
RCT/Quantitative Analysis	*n* = 8 (4 M; 4 F) Federated athletes (including basketball, volleyball, handball, and athletics) with patellar tendinopathy.	To analyze the effect of 4 weeks of physical rehabilitation with HMB supplementation in athletes diagnosed with patellar tendinopathy.	Athletes were assigned to receive HMB (*n* = 4) or placebo (*n* = 4) during 4 weeks. Body composition, perceived pain, and muscular function were measured.	3 g of HMB per day 60 min before exercise.	HMB supplementation might enhance muscle power in athletes with patellar tendinopathy. It seems to optimize adaptions during the non-invasive treatment of the injury.	Sánchez-Gómez et al. (2022) [59]
Narrative Review/Qualitative Analysis	77 references (from year of publication to 2022).	To define the proper nutritional elements tailored by athletes’ needs in order to facilitate their fast return to sports after surgery or injury.	Expert view and non-structured analysis of the scientific literature.	Adequate energy intake (25–30 kcal/kg/day). Adequate intake of carbohydrates and especially proteins (type, frequency, and amount). CrM, Fish oil (Ω3), curcumin, bromelain. Avoid deficiencies in vitamins and minerals.	Diets that include high quality nutrients, rich in macro, micro, and bioactive compounds are recommended. Biomedical indices and vitamin and mineral levels should be evaluated and monitored to avoid deficiencies.	Papadopoulou et al. (2022) [10]
Narrative Review/Qualitative Analysis	182 references (from year of publication to 2022).	To present various nutritional strategies for reducing the risk of injury and improving the treatment and rehabilitation process in combat sports.	Expert view and non-structured analysis of the scientific literature.	Maintain energy availability (45 kcal per kg of FFM per day). High protein diet (2 to 2.3 g/kg/day) with 20–25 g per meal of leucine-rich protein (3 g). 15 g of COL 60 min before an exercise rehabilitation program. CrM, Ω3, vitamin D, and Ca.	It is important to provide athletes with an adequate amount of macro- and micro-nutrients and nutritional supplements to meet the demands of the catabolic state and contribute to the injury-healing process.	Turnagöl et al. (2022) [46]
Scoping Review/Qualitative Analysis	155 references (from year of publication to 2022).	To evaluate current research on the use of nutritional supplements for treating tendon injuries.	Structured literature search in Medline, Cinahl, Amed, EMBase, SPORTDiscus, and Cochrane. Sixteen references met the inclusion criteria.	COL, hydrolyzed COL, amino acids, vitamin C, glucosamine, HMB, Ω3, antioxidants, and CrM have been studied.	Certain nutritional supplements might have pain relieving, anti-inflammatory, and structural tendon effects that augment the positive functional outcomes gained from progressive exercise rehabilitation.	Burton et al. (2022) [45]
Cross-sectional Study/Quantitative Analysis	*n* = 133 (77 M; 56) International level Australian rowers from seniors (*n* = 115) and under 23-year-old levels (*n* = 18).	To identify nutrition-related factors associated with a history of rib stress injuries in elite rowers (including the injury time course).	Online questionnaire for historical records and a qualified dietitian collected information regarding habitual Ca intake. Body composition and BMD were measured with DXA. A sub-group of participants (*n* = 68) were assessed for vitamins D and K.	NA	Nutritional strategies to support injury prevention should focus on energy availability and its contribution to health and function, including menstrual status.	Lundy B et al. 2022 [44]

Ω3: Omega-3 fatty acids; ACL: Anterior cruciate ligament; BCAA: Branched chain amino acids; BMD: Bone mineral density; Ca: Calcium; CFU: Colony-forming units; COL: Collagen peptides and specific gelatin products; CoQ10: Coenzyme Q10 (ubiquinone); CrM: Creatine monohydrate; Cu: Copper; DASH: Disabilities of the Arm, Shoulder and Hand; EAA: Essential amino acids; FFM: Fat-free mass; HMB: β-hydroxi-β-methylbutyrate; IU: International units; Mn: Manganese; RCT: Randomized controlled trial; DXA: Dual-energy X-ray absorptiometry; VAS: Visual analogue scale; Zn: Zinc.

## Data Availability

Not applicable.
